# Increased Type I Interferon Activity with Concurrent Plasmablast Expansion Identifies Systemic Lupus Erythematosus Patients with Poor Outcomes

**DOI:** 10.3390/ijms27062852

**Published:** 2026-03-21

**Authors:** Konrad Speidel, Qingyu Cheng, Laleh Khodadadi, Benedikt Sinzinger, Jonas Martin, Anne E. Beenken, Robert Biesen, Gerhard Krönke, Falk Hiepe, Tobias Alexander

**Affiliations:** 1 Department of Rheumatology and Clinical Immunology, Charité—Universitätsmedizin Berlin, 10117 Berlin, Germany; konrad.speidel@charite.de (K.S.); jonas.martin@charite.de (J.M.);; 2 Deutsches Rheuma-Forschungszentrum Berlin, a Leibniz Institute, 10117 Berlin, Germany; 3 Department of Nephrology and Medical Intensive Care, Charité—Universitätsmedizin Berlin, 10117 Berlin, Germany

**Keywords:** B cells, interferon, plasma cells, SIGLEC-1, SLE

## Abstract

Recent evidence suggests that type I interferon (IFN) activity has prognostic relevance in systemic lupus erythematosus (SLE). This study investigated whether combining IFN activity with elevated peripheral blood plasmablast (PB) levels—another key feature of lupus pathophysiology—improves risk stratification for poor clinical outcomes. Clinical data were prospectively collected at a single lupus center. Flow cytometry was performed on freshly isolated peripheral blood mononuclear cells to investigate Sialic acid-binding Immunoglobulin-like Lectin 1 (SIGLEC-1) as a surrogate marker of IFN activity, alongside CD19^+^CD20^−^CD27^high^HLA-DR^+^ PB frequencies. A total of 1276 samples from 121 patients were analyzed. At baseline, 48.8% of patients exhibited high IFN activity, including 27.3% with concurrent elevation in IFN and PB activity and 21.5% with isolated IFN activity. Patients with simultaneous IFN and PB activity showed higher anti-dsDNA antibody levels, were less frequently in DORIS remission (24.2% vs. 50.0%) and required higher daily prednisolone dosages (6.3 vs. 2.0 mg) than those with isolated IFN activity. During a median follow-up of 4.5 years (range 0.8–6.6), these patients experienced more flares (132 vs. 54, OR 1.42), required longer to achieve remission (median 399 vs. 140 days), and had a higher median time-adjusted prednisolone dose (5.6 vs. 3.0 mg). Concurrent elevation in IFN and PB activity identifies SLE patients with a poorer prognosis compared to isolated IFN activity. These findings suggest that combined IFN and PB assessment may improve prognostic stratification and support personalized treatment strategies in SLE.

## 1. Introduction

Systemic lupus erythematosus (SLE) is a chronic autoimmune disease characterized by a wide range of clinical manifestations and immunological disturbances, including elevated type I interferon (IFN) activity and increased frequencies of peripheral blood plasmablasts (PBs) [1]. The early use of biologic therapies targeting IFN receptor or B cell activating factor (BAFF) has improved disease control, and the implementation of a treat-to-target (T2T) strategy has provided significant protection against damage accrual [2]. However, disease relapses remain common in SLE and are associated with increased morbidity and mortality [3]. Therefore, effective risk stratification models are essential for the early identification of patients at risk of unfavorable long-term outcomes.

Type I interferons represent a central component of innate antiviral immunity and are increasingly recognized as key mediators of autoimmunity. The interferon family comprises three major classes. Type I interferons include multiple IFN-α subtypes, as well as IFN-β, IFN-ε, IFN-κ and IFN-ω, which signal through the heterodimeric interferon-α/β receptor composed of IFNAR1 and IFNAR2. Type II interferon is represented solely by IFN-γ and signals through the IFNGR1/IFNGR2 receptor complex, while type III interferons (interferon-λ 1–4) signal through a receptor composed of IFNLR1 and IL-10R2 and exert antiviral effects, particularly at epithelial barrier surfaces [4,5]. Type I interferons are produced predominantly by plasmacytoid dendritic cells following activation of nucleic-acid-sensing pattern recognition receptors such as Toll-like receptors (TLR7 and TLR9) or cytosolic nucleic acid sensors, including the cGAS-STING pathway [4,5].

Multiple factors influence the magnitude and persistence of type I interferon activity. Viral infections represent classical triggers of IFN production, but endogenous nucleic acids released during apoptosis or NETosis can activate the same sensing pathways and promote chronic IFN signaling in autoimmune diseases such as SLE [5,6]. Genetic variants affecting nucleic acid sensing pathways, impaired clearance of apoptotic debris and immune complex formation further contribute to sustained interferon pathway activation [5]. Environmental and physiological factors may additionally modulate IFN responses. Experimental studies suggest that psychological or physiological stress can influence IFN signaling through neuroendocrine-immune interactions involving activation of the hypothalamic–pituitary–adrenal axis and sympathetic nervous system pathways that affect cytokine production and immune cell activation [7,8].

Several diagnostic approaches have been developed to quantify IFN activity in clinical and research settings. Direct measurement of circulating interferon proteins can be performed using enzyme-linked immunosorbent assays or ultrasensitive digital immunoassays such as single-molecule array (Simoa) technology, which enables detection of extremely low concentrations of IFN-α in serum [6,9]. Alternatively, IFN pathway activation can be assessed indirectly through the expression of IFN-stimulated genes using transcriptomic approaches or quantitative PCR-based IFN-scores, which have been widely applied in SLE and other autoimmune diseases [6,10]. Flow-cytometric detection of IFN-inducible surface molecules, including Sialic acid-binding Immunoglobulin-like Lectin 1 (SIGLEC-1) expression on monocytes, has also emerged as a clinically applicable biomarker reflecting type I interferon activity in peripheral blood [11,12].

Beyond their diagnostic utility, interferons have long been used therapeutically in medicine. Recombinant interferons are employed in the treatment of several viral infections and malignancies, including chronic viral hepatitis and certain hematologic cancers [4]. Conversely, excessive activation of IFN pathways contributes to the pathogenesis of autoimmune diseases such as SLE, which has led to the development of targeted therapies blocking IFN signaling, including monoclonal antibodies directed against the IFN-α/β receptor such as anifrolumab [5,13].

Initial cross-sectional studies demonstrated a strong correlation between IFN-activity and an increased overall disease activity [10,12]. Although longitudinal analyses indicated that IFN scores show little change over time [14], subsequent studies have shown that upregulated IFN is associated with a higher risk of flares [9,11] and a reduced likelihood of achieving Lupus Low Disease Activity State (LLDAS) [15]. Similarly, the expansion of PB in peripheral blood is a putative biomarker of lupus severity and may predict the risk of disease flares [16,17]. While recent studies have reported a strong overlap between IFN activity and PB expansion [18], particularly during active disease, longitudinal analysis examining these markers in combination is lacking.

Based on the assumption that a better understanding of the dynamics of IFN activity and peripheral blood PB frequencies may translate into novel risk stratification models for personalized medicine in SLE, we now investigated Sialic Acid–Binding Ig-like Lectin 1 (SIGLEC-1)—as a surrogate marker of IFN [12,19]—alongside the frequencies of CD19^+^CD20^−^CD27^high^HLA-DR^+^ plasmablasts longitudinally in a single-center cohort of SLE.

## 2. Results

### 2.1. Segregation of IFN Activity and PB Frequencies by Flow Cytometry

**Figure 1:**
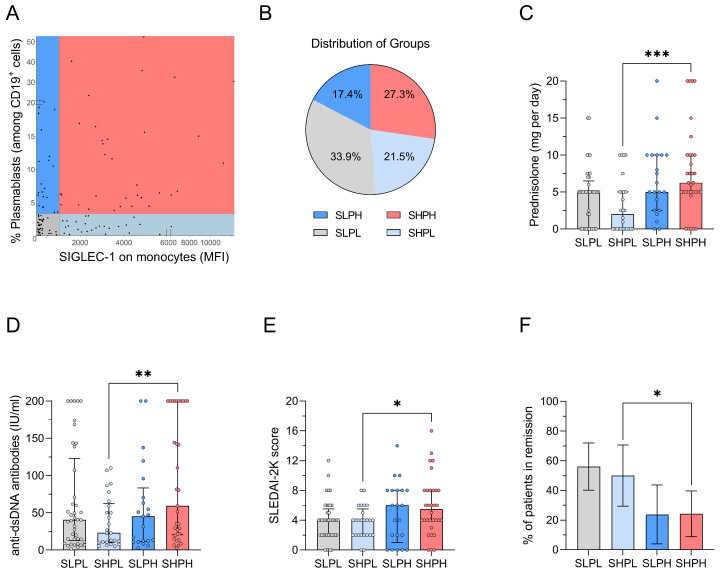
Baseline characteristics of 121 SLE patients. (**A**) Scatterplot outlining SIGLEC-1 expression on monocytes versus PB among CD19^+^ B cells, determined by flow cytometry. Each dot represents one patient. (**B**) Distribution of patients according to their PB and IFN activity state. (**C**–**E**) Daily prednisolone doses (mg), serum anti-dsDNA antibody titers (U/L), and SLEDAI-2K scores at baseline. Bars represent group medians, with individual data points (dots) corresponding to single patients. (**F**) Percentage of patients in DORIS-remission. Error bars represent 95% confidence intervals. Statistical analysis was performed using the Mann–Whitney test for continuous variables and the Chi-squared test for categorical variables. * *p* < 0.05; ** *p* < 0.01; *** *p* < 0.001. MFI: median fluorescence intensity. SLEDAI-2K: Systemic Lupus erythematosus activity score 2000. SLPL: SIGLEC-1^low^ plasmablast^low^. SHPL: SIGLEC-1^high^ plasmablast^low^. SLPH: SIGLEC-1^low^ plasmablast^high^. SHPH: SIGLEC-1^high^ plasmablast^high^.

A total of 121 patients with SLE were included in this prospective analysis. Their median disease duration was 5 years (range 0–33) and the median SLEDAI-2K score was 4 (range 0–16). Detailed baseline characteristics are provided in Table S1. All patients underwent longitudinal sample analysis (median: 9 samples, range: 5–36), yielding a total of 1276 measurements over a study period of 4.5 years (range 0.8–6.6 years). At baseline, patients were categorized into four distinct cohorts based on their initial IFN and PB activity states, as determined by flow cytometry (Figure 1A). Overall, 48.8% of patients were IFN-high, and 27.3% exhibited both elevated IFN and PB activity, while 21.5% showed increased IFN activity alone, and 33.9% exhibited neither (Figure 1B). Detailed patient characteristics stratified by IFN and PB status are summarized in Table 1.

### 2.2. Concurrent IFN and PB Activity Is Associated with High Disease Activity

To determine the added value of assessing PB activity in addition to IFN status for predicting disease severity, we compared baseline demographic characteristics and clinical manifestations between patients with isolated IFN activity and those with concurrent IFN/PB activity. We found no significant differences in age, sex, ethnicity, disease duration or number and type of affected organs between these groups (Table 1). However, patients with elevated IFN and PB activity at baseline required significantly higher doses of daily prednisolone (Figure 1C; median 6.3 vs. 2.0 mg, *p* = 0.001), had higher serum anti-dsDNA antibody titers (Figure 1D; median 59.4 vs. 23.1 U/L, *p* = 0.010) and higher SLEDAI-2K scores (Figure 1E; median 5.5 vs. 4.0, *p* = 0.030), and were less frequently in DORIS remission (Figure 1F; 24.2% vs. 50.0%, *p* = 0.043). The groups did not differ as regards the presence of anti-ENA (extractable nuclear antigens) antibodies in serum or the underlying treatment with regard to immunosuppressive and biologic therapies (Table 1).

### 2.3. Stability of IFN Activity over Time

**Figure 2:**
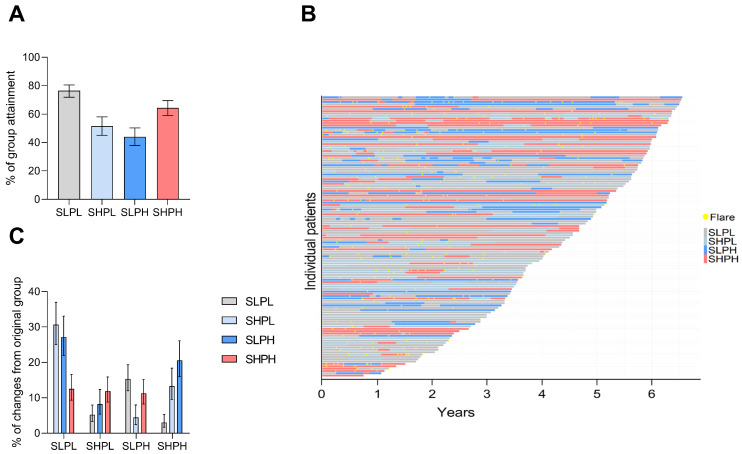
Changes in IFN and PB activity state over time. (**A**) Proportion of patients with stable group assignment over time. Error bars represent 95% confidence intervals. (**B**) Individual changes in group assignment over time. (**C**) Proportion of patients with transition into other group assignment over time. Error bars represent 95% confidence intervals.

We next assessed the longitudinal stability of patients’ initial group assignments over time. Overall, there was limited fluctuation. Patients with concurrent IFN and PB activity at baseline maintained this state in 67.8% of all subsequent follow-up assessments over a median follow-up duration of 4.5 years (range 0.8–6.6) and a median of 9 (range 4–23) follow-up visits (Figure 2A). Stability was lower in patients with isolated IFN activity at baseline (57.3%) and higher in those with neither IFN nor PB activity (77.3%). The proportion of patients constantly remaining in their initial group assignment was substantial lower (Supplementary Figure S1).

Changes in group assignment at the individual level are provided in Figure 2B. Changes from concurrent IFN/PB activity into another group are similarly distributed (Figure 2C). Notably, patients initially classified as IFN-low with high PB activity were the most likely to change group assignment over time, suggesting a transitional phase with subsequent change into either IFN/PB-low or IFN/PB-high states. Detailed changes in group assignment are provided in Supplementary Figure S2.

### 2.4. Achieving Remission Is Prolonged in Patients with Concurrent IFN and PB Activity States

**Figure 3:**
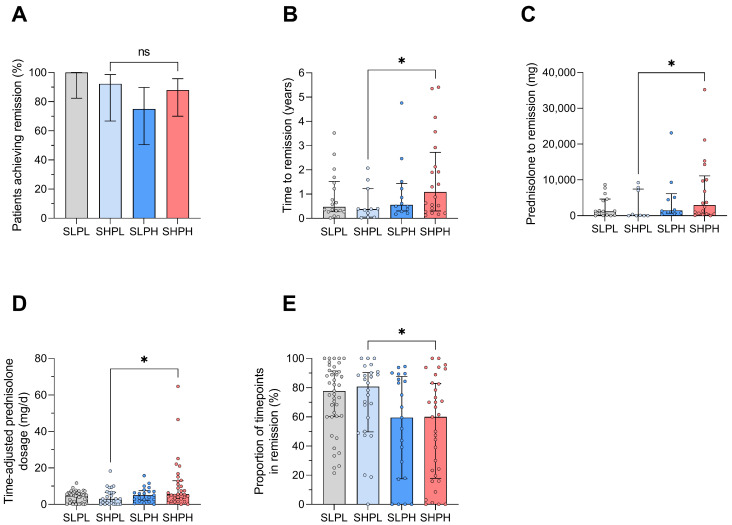
Probability of achieving remission. (**A**) Percent of patients achieving remission during follow-up according to baseline group assignments (SHPH n = 25; SHPL n = 13; SLPH n = 16; SLPL n = 18). Statistical analysis was performed using the Chi-square test (*p* = 0.681). Error bars represent 95% confidence intervals. (**B**) Time to first remission (years) per patient who were not in remission at baseline. Bars represent group medians, with individual data points (dots) corresponding to single patients. (**C**) Cumulative prednisolone dose (mg) required to reach remission. Bars represent group medians, with individual data points (dots) corresponding to single patients. (**D**) Time-adjusted daily prednisolone dose according to baseline IFN/PB activity group in all patients. Bars represent group medians, with individual data points (dots) corresponding to single patients. (**E**) Percentage of follow-up visits in which patients were in remission throughout the follow-up period. Bars represent group medians, with individual data points (dots) corresponding to single patients. Statistical analysis was performed with Mann–Whitney U test. ns *p* ≥ 0.05; * *p* < 0.05.

We next examined the probability of maintaining or achieving remission according to patients’ initial group assignment. Patients who were in remission at their initial visit generally maintained remission throughout follow-up, with no significant differences observed between groups (Supplementary Figure S3). Likewise, patients who were not in remission at baseline achieved remission during follow-up regardless of their initial assignment (Figure 3A). However, the time to achieve remission differed significantly between groups. Patients initially assigned to the SHPH group required a longer time to achieve remission compared with those with isolated IFN activity (median 1.1 vs. 0.4 years, *p* = 0.040) (Figure 3B). Furthermore, these patients required higher cumulative prednisolone doses to achieve remission (2989 vs. 80 mg, *p* = 0.049) (Figure 3C). Consequently, patients with concurrent IFN and PB activity at baseline received higher time-adjusted prednisolone doses (5.6 vs. 3.0 mg per day, *p* = 0.010) (Figure 3D) and spent a smaller proportion of the observational period in remission (60% vs. 81%, *p* = 0.040) (Figure 3E).

### 2.5. Increased Risk of Flare Development in Patients with Concurrent IFN and PB Activity States

**Figure 4:**
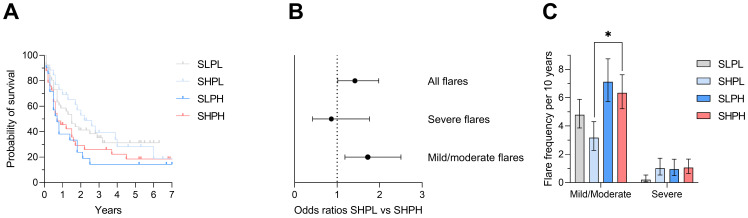
Flare development during follow-up. (**A**) Kaplan–Meier curves showing flare-free survival during follow-up. (**B**) Forrest plot displaying odds ratios (OR) for flare development comparing SHPL with SHPH during follow-up. SHPL was used as the reference group (OR > 1 indicates higher odds in SHPH). Any flare: OR 1.42 (95% CI 1.02–1.98); mild/moderate flare: OR 1.72 (CI 1.18–2.50); severe flare: OR 0.86 (CI 0.42–1.76). The dotted line indicates an odds ratio of 1 (no effect). (**C**) Flare frequency per 10 years according to group. Comparison between SHPL and SHPH was performed using a two-tailed Mann–Whitney U test: *p* = 0.030. Error bars represent 95% confidence intervals. * *p* < 0.05.

We finally investigated the flare rate during the study period measuring SFI. Kaplan–Meier curves for flare-free survival are provided in Figure 4A. Overall, flares were more frequent in patients initially assigned to the IFN/PB activity group compared to those with IFN activity alone (132 vs. 54 flares, OR 1.42, CI 1.02–1.98, Figure 4B). Most of the flares were mild to moderate, with even higher odds ratios for flare development in the IFN/PB group (113 vs. 41 flares, OR 1.72, CI 1.18–2.50). Finally, the time-adjusted mild-to-moderate flare frequency occurring in the subgroup of patients with initial IFN/PB activity state compared to those with IFN activity alone was significantly higher (median 6.3 vs. 3.3 flares per 10 years, *p* = 0.030), as shown in Figure 4C.

## 3. Discussion

In this single-center, prospective study, we evaluated the prognostic relevance of combined type I interferon activity, assessed by SIGLEC-1 expression on monocytes, and peripheral blood plasmablast (PB) levels in patients with SLE. Our findings demonstrate that patients exhibiting concurrent elevated IFN activity and PB frequencies experience a less favorable clinical course compared with those showing isolated IFN activity. Specifically, these patients presented with higher disease activity, as reflected by increased SLEDAI-2K scores, elevated anti-dsDNA titers, a greater requirement for daily prednisolone, and lower DORIS-remission rates. This association occurred despite similar demographic and organ involvement profiles, suggesting that concurrent IFN/PB activation identifies a biologically distinct and clinically relevant subgroup of patients. While previous studies have demonstrated that elevated IFN activity alone is associated with a less favorable disease course in SLE [20], our combined assessment of IFN activity and PB frequencies further refines this stratification by identifying a subset of IFN-positive patients with particularly unfavorable clinical trajectories.

Longitudinally, the overall probability of achieving or maintaining remission did not differ according to baseline IFN and PB status, but important differences in disease dynamics emerged. Patients with concurrent IFN/PB activity experienced higher flare rates and required a longer time to achieve remission. Consequently, they spent less cumulative time in remission and required higher time-adjusted glucocorticoid exposure, both of which are established risk factors for cumulative organ damage accrual [2,21]. These findings suggest that simultaneous activation of IFN-driven immune pathways and humoral immune responses identifies a subgroup of patients with more persistent, relapse-prone and treatment-intensive disease. In contrast, patients lacking IFN and PB activity demonstrated the most favorable outcomes, underscoring the potential utility of these biomarkers in defining high- and low-risk phenotypes and refining prognostic stratification.

Approximately half of the patients in our cohort exhibited increased IFN activity, consistent with previous reports [15]. Moreover, the strong correlation observed between SIGLEC-1 expression and disease activity aligns with earlier studies identifying SIGLEC-1 as a reliable surrogate marker of interferon activity in SLE [11,12,19]. However, in contrast with previous reports demonstrating robust associations between IFN activity and PB frequencies [16,18,22] or B cell activating factor (BAFF) transcript levels [23,24,25], we observed that only 50% of patients with elevated IFN activity also exhibited increased PB frequencies. These findings are in line with recent data describing distinct patterns of IFN upregulation and B and plasma cell dysregulation in lupus nephritis [26], supporting the concept that immunologically defined patient subgroups exist within IFN-high populations.

This notion is further supported by transcriptomic analyses identifying a distinct plasma cell gene expression signature in autoimmune diseases, including SLE, which reflects antibody-secreting cell activity and only partially overlaps with interferon-related immune signatures, highlighting the presence of multiple, partially independent immunological pathways within SLE [27].

Our longitudinal analyses revealed that IFN/PB states were relatively stable over time, consistent with previous reports investigating type 1 interferon in SLE [14]. Particularly, patients with concurrent IFN/PB activity at baseline maintained this state at 67.8% of follow-up assessments. Patients with isolated IFN or PB activity showed greater fluctuation, while those lacking IFN/PB activity remained stable most consistently. These findings support the notion that simultaneous IFN and B cell activation represents a rather stable immune signature in a subset of SLE patients, whereas isolated elevations may reflect a more transient or context-dependent state.

Mechanistically, our findings are consistent with the well-established role of type 1 interferons in driving PB differentiation [23,28] and autoantibody production [23,28]. The combined presence of IFN-driven innate immune activation and enhanced B cell activity may therefore perpetuate autoimmunity, resulting in higher disease activity and treatment burden. Assessment of SIGLEC-1 as a surrogate for IFN activity alongside PB frequencies provides a practical means to capture this dual-pathway activation in peripheral blood.

Our study has important clinical implications. Integrating type 1 interferon and PB assessment into routine patient monitoring may enable earlier identification of individuals at risk of persistent, relapse-prone disease who may benefit from intensified immunomodulatory therapy or closer follow-up. Conversely, patients with low IFN/PB activity may be considered for treatment de-escalation strategies. Furthermore, the stability of IFN/PB activation status suggests that this dual-marker approach could serve as a reliable stratification tool over time.

Despite providing novel insights, our study has several limitations. First, the cohort was relatively small and heterogenous with respect to organ manifestations and treatment regimens, which may limit the generalizability of our findings to the broader SLE population. Second, the open-label, single-center design introduces potential sources of bias. Third, this study was initiated before the availability of anifrolumab, a biologic targeting IFN receptor, limiting our ability to predict responses to such targeted therapy. Finally, our longitudinal analysis focused on disease activity, flare incidence and remission rates, but did not include measures of damage accrual. Future research should aim to validate these biomarkers in larger, multi-center cohorts and evaluate whether interventions targeting interferon pathways or B cell function can improve outcomes in patients identified as high-risk based on combined SIGLEC-1 and PB assessments. Additionally, investigating the temporal dynamics of these biomarkers in relation to disease activity and treatment responses could provide further insights into their roles in SLE pathogenesis.

In conclusion, our study demonstrates that the combination of increased interferon activity and elevated peripheral blood PB is associated with poorer clinical outcomes in SLE patients. This dual biomarker approach holds promise for enhancing prognostic assessments and guiding more tailoring therapeutic strategies in SLE management.

## 4. Materials and Methods

### 4.1. Patients

Consenting adult patients meeting the 2012 Systemic Lupus International Collaborating Clinics (SLICC) Classification Criteria for SLE [29] were recruited from the Charité—University Medicine Berlin between January 2015 and September 2021. Heparinized peripheral blood was collected for biomarker analysis at the time of the patients’ routine investigation. Clinical data were prospectively collected relating to the blood sampling, including demographic data, medication use, the SLE disease activity index (SLEDAI-2K) [30], the Safety of Estrogens in Lupus Erythematosus: National Assessment (SELENA)-SLEDAI Flare Index (SFI) [31], and rates of DORIS remission [32] or LLDAS [33].

### 4.2. Cell Isolation and Flow Cytometry

Peripheral blood mononuclear cells were isolated from heparinized blood using density centrifugation (Ficoll-Paque PLUS, Cytiva, Wilmington, DE, USA). Flow cytometry was performed on freshly isolated PBMCs. Cells were stained 10 min at 4 °C with the antibodies indicated in Supplementary Table S2. DAPI was added to stain dead cells, and samples were acquired on a FACSCanto II (BD Biosciences, San Jose, CA, USA). FACS analysis was performed following the guidelines for the use of flow cytometry and cell sorting in immunological studies [34]. IFN activity was measured by assessing the surface expression of SIGLEC-1 (mean fluorescence intensity) on CD14^+^ monocytes, as recently described [12]. PBs were determined by analyzing the frequency of CD14^−^CD3^−^DAPI^−^CD19^+^CD20^−^CD27^++^HLA-DR^+^ cells among CD19^+^ B cells. IFN activity was measured by flow cytometry of freshly isolated PBMC by assessing the surface expression of SIGLEC-1 (mean fluorescence intensity) on CD14^+^ monocytes, as recently described [12]. Cytometry data were analyzed using FlowJo v10.6.2 (FlowJo, LLC, Ashland, OR, USA). The gating strategy of flow cytometry experiments is illustrated in Supplementary Figure S4.

### 4.3. Statistical Analysis

Statistical analysis was performed using R (Version 2022.07.1+554) and GraphPad Prism (Version 9.4.1) software. Mann–Whitney U tests were used to compare groups of non-parametric continuous data. Chi-squared tests were used for categorical data. Odds ratios for key baseline variables were estimated with univariable logistic regression and are displayed with 95% CIs. Separately, mild/moderate and severe flare incidences were calculated for each patient as events per 10 patient years of follow-up and compared between groups with non-parametric statistics, as detailed above. *p* values of <0.05 were considered statistically significant.

## Figures and Tables

**Table 1:** Demographics and clinical characteristics of investigated SLE patients. Statistical analysis was performed to compare data between the IFN^low^PB^high^ and IFN^high^PB^high^ groups. Categorical variables were compared using the χ^2^ test, ordinal variables were analyzed using the Mann–Whitney U test.

Variable	IFN^low^PB^low^ (n = 41)	IFN^low^PB^high^ (n = 21)	IFN^high^PB^low^ (n = 26)	IFN^high^PB^high^ (n = 33)	*p*-Value
Age, median (range), years	49 (26–75)	43 (31–69)	45 (29–84)	43 (28–88)	0.783
Female, n (%)	36 (87.8)	18 (85.7)	23 (88.5)	31 (93.3)	0.780
Disease duration, median (range), years	6.0 (0–30)	4.0 (0–33)	3.5 (0–29)	5.0 (0–28)	0.429
SLEDAI-2K, median (range)	4.0 (0–12)	6.0 (0–14)	4.0 (0–10)	5.5 (0–16)	0.030
LLDAS, n (%)	26 (63.4)	7 (33.3)	16 (61.5)	12 (36.4)	0.058
Remission, n (%)	23 (56.1)	5 (23.8)	13 (50.0)	8 (24.2)	0.043
Affected organ systems, median (range)	2 (0–5)	2 (0–4)	2 (1–4)	2 (1–6)	1.000
Musculoskeletal involvement, n (%)	35 (85.4)	15 (71.4)	25 (96.2)	27 (81.8)	0.199
Mucocutaneous involvement, n (%)	29 (70.7)	16 (76.2)	22 (84.6)	25 (75.8)	0.608
Renal involvement, n (%)	17 (41.5)	10 (47.6)	8 (30.8)	13 (39.4)	0.680
Hematological involvement, n (%)	8 (19.5)	2 (9.5)	5 (19.2)	12 (36.4)	0.249
Cardiorespiratory involvement, n (%)	5 (12.2)	7 (33.3)	2 (7.7)	8 (24.2)	0.183
Neuropsychiatric involvement, n (%)	5 (12.1)	3 (14.3)	2 (7.7)	3 (9.1)	1.000
Prednisolone use, n (%)	29 (70.7)	19 (90.5)	15 (57.7)	29 (87.9)	0.020
Prednisolone dose, median (range), mg/day	5.0 (0–15)	5.0 (0–20)	2.0 (0–10)	6.3 (0–20)	0.001
Hydroxychloroquine, n (%)	33 (80.5)	15 (71.4)	15 (57.7)	25 (75.8)	0.233
Azathioprine, n (%)	11 (26.8)	7 (33.3)	3 (11.5)	9 (27.3)	0.244
Methotrexate, n (%)	3 (7.3)	2 (9.5)	6 (23.1)	2 (6.1)	0.130
Mycophenolate mofetil, n (%)	10 (24.4)	2 (9.5)	6 (23.1)	5 (15.2)	0.660
Belimumab, n (%)	5 (12.2)	3 (14.3)	2 (7.7)	2 (6.1)	1.000
Anti-dsDNA titer, median (range), U/L	40.6 (1.7–200)	45.3 (2.8–200)	23.1 (5.3–200)	59.4 (2.8–200)	0.010
C3, median (range), mg/L	890 (600–1510)	920 (510–1590)	910 (530–1140)	800 (190–1300)	0.059
C4, median (range), mg/L	180 (40–360)	180 (60–330)	135 (40–260)	140 (20–290)	0.885
C3 and/or C4 consumption, n (%)	22 (53.7)	10 (47.6)	14 (53.8)	24 (72.7)	0.219

## Data Availability

The data are not publicly available due to privacy and ethical constraints related to sensitive clinical data in a rare disease cohort. Anonymized data may be made available by the corresponding author upon reasonable request and subject to institutional approval.

## Supplementary Materials

The following supporting information can be downloaded at: https://www.mdpi.com/article/10.3390/ijms27062852/s1.
