# Supplementary material for: Increased Type I Interferon Activity with Concurrent Plasmablast Expansion Identifies Systemic Lupus Erythematosus Patients with Poor Outcomes
Source: Int J Mol Sci. 2026 Mar 21;27(6):2852. doi: 10.3390/ijms27062852 (PMC13026582; doi:10.3390/ijms27062852)
Supplement: Supplementary file 1 [file ijms-27-02852-s001.zip › ijms-4202892-supplementary.pdf]

Supplementary Figures and Tables

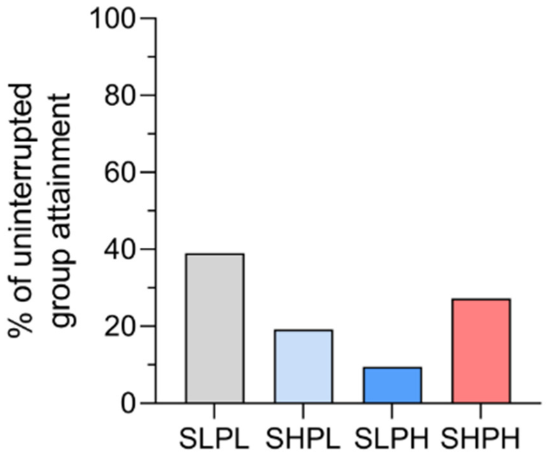

Figure S1: Proportion of patients who constantly maintained assignments to their baseline IFN/PB activity group throughout the entire observation period. For each initial group (SLPL, SHPL, SLPH, SHPH), bars represent the percentage of individuals whose longitudinal measurements consistently remained within their original group without any transition during follow-up.

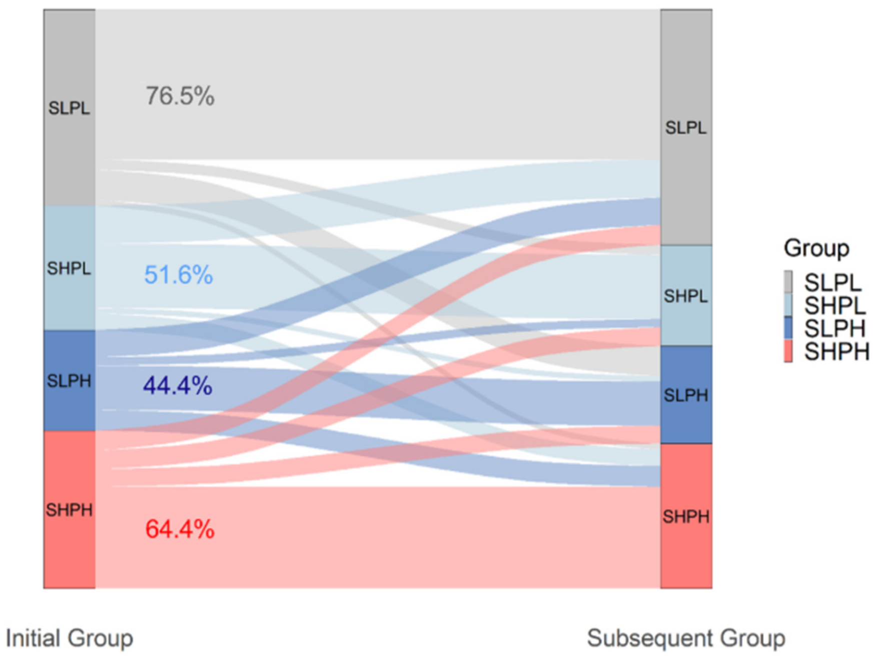

Figure S2. Sankey plot showing longitudinal transitions between IFN/PB activity groups. The left side represents baseline group assignment (width proportional to the number of patients in each baseline group), and the right side represents the distribution of all subsequent follow-up measurements (width proportional to the total number of follow-up measurements assigned to

each group). Flows illustrate transitions from the baseline group to subsequent group classifications, with flow width corresponding to the number of follow-up measurements. The percentage shown within each major same-group flow indicates the proportion of subsequent follow-up measurements that remained in the respective baseline group.

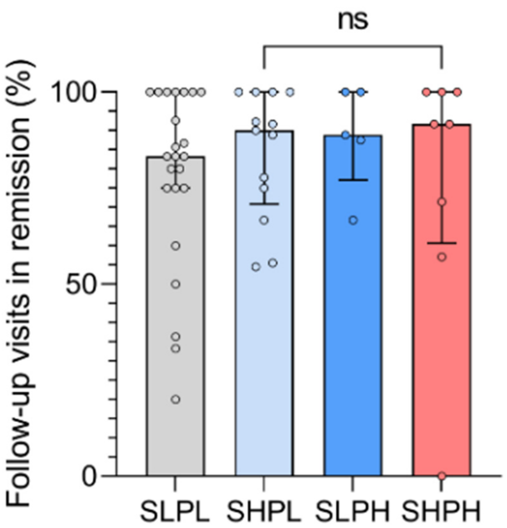

Figure S3. Proportion of time-points in which patients who were in remission at baseline maintained remission during follow-up. Median/IQR ranges are shown, each point represents one patient (SLPL n=23; SHPL n=13; SLPH n=5; SHPH n=8). Statistical comparison between SHPL and SHPH was performed using the Mann–Whitney U test and showed no significant difference (P = 0.970).

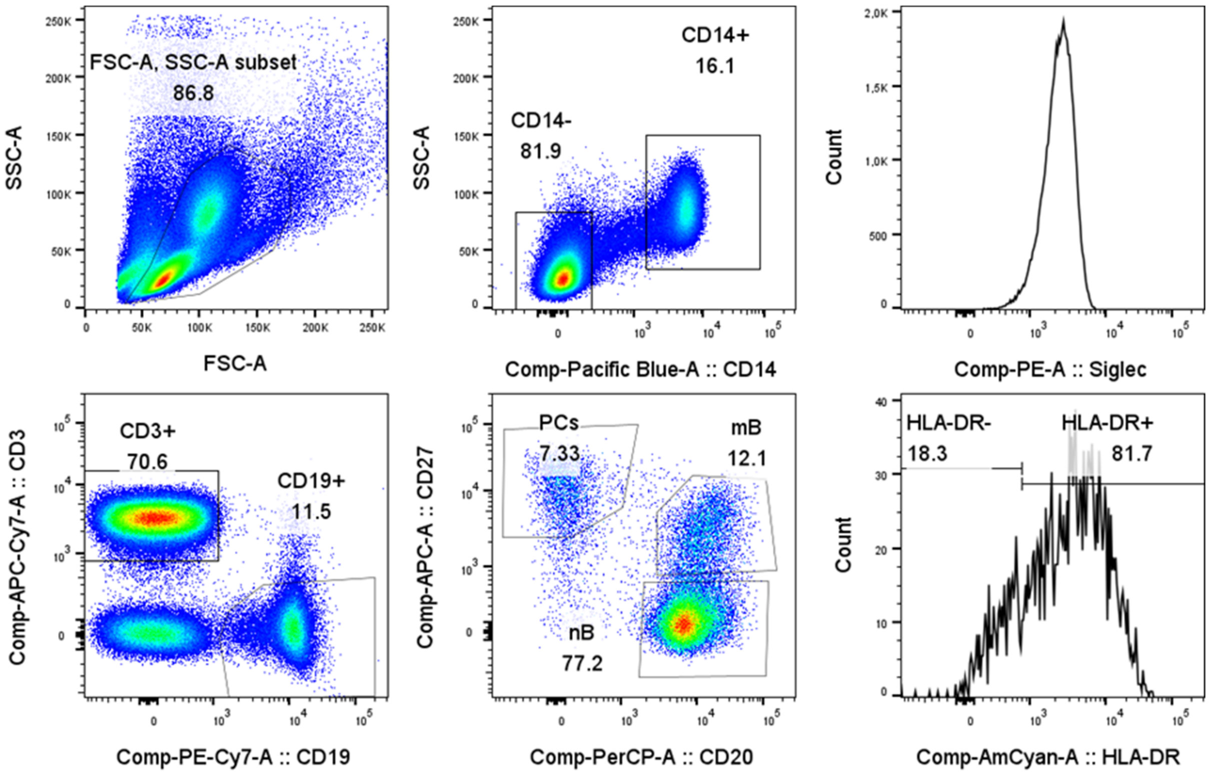

Figure S4. Flow-cytometric gating for PB and SIGLEC-1. (A) PB among $CD19^+$ B cells: live ($DAPI^-$), $CD3^-CD14^-$, $CD19^+$ B cells → PB defined as $CD20^-CD27^{++}HLA\text{-}DR^+$; frequency reported as % of $CD19^+$. (B) SIGLEC-1 on monocytes: $CD14^+$ monocytes gated and SIGLEC-1 (mean fluorescence intensity, MFI) quantified on this gate.

**Characteristic**	**All Patients**
Number of patients (n, %)	121
Age (median, range)	46 (26–88)
Female (n, %)	108 (89.3%)
Disease duration in years (median, range)	5.0 (0.0–33.0)
SLEDAI-2K (median, range)	4.0 (0.0–16.0)
LLDAS (n, %)	61 (50.4%)
Remission (n, %)	49 (40.5%)
Number of affected organ systems (median, range)	2 (0–6)
Clinical manifestations (n, %)	
Musculoskeletal	102 (84.3%)
Mucocutaneous	92 (76.0%)
Renal	48 (39.7%)
Haematological	27 (22.3%)
Cardiorespiratory	22 (18.2%)
Neuropsychiatric	13 (10.7%)
Therapies (n, %)	
Prednisolone	91 (75.2%)
Hydroxychloroquine	87 (73.1%)
Azathioprine	29 (24.9%)
Methotrexate	13 (10.9%)
Mycophenolate mofetil	23 (19.3%)
Belimumab	12 (9.9%)
Prednisolone dose mg/day (median, range)	5.0 (0.0–20.0)
Extractable nuclear antigens (median, range)	1.0 (0.0–4.0)
Unique extractable nuclear antigens (n, %)	
Anti-SSA/Ro antibody	52 (43.7%)
Anti-SSB/La antibody	16 (13.4%)
Anti-U1RNP antibody	24 (20.2%)
Anti-RNP70 antibody	18 (15.1%)
Anti-Smith antibody	18 (15.1%)
Anti-Centromere antibody	1 (0.8%)
Anti-dsDNA titre U/L (median, range)	40.2 (1.7–200.0)
C3 titre mg/L (median, range)	860 (190–1590)
C4 titre mg/L (median, range)	165 (20–360)
Patients with consumption of C3 and/or C4 complement (n, %)	70 (57.9%)

Table S1. Baseline characteristics of the overall study population. The table summarizes demographic, clinical, serological, and treatment-related parameters of all included patients (n = 121), representing the combined cohort of the four study groups. Continuous variables are presented as median (range), and categorical variables as number (percentage).

Antibody / Reagent	Fluorochrome	Clone	Company	Cat. No.	Dilution/Test
Anti-human IgD	FITC	IA6-2	BioLegend	348206	1:20
Anti-human CD169 (Siglec-1)	PE	7-239	BioLegend	346004	1:20
Anti-human CD20	PerCP	2H7	BioLegend	302324	1:20
Anti-human CD19	PE/Cy7	HIB19	BioLegend	302216	1:20
Anti-human CD27	APC	O323	BioLegend	302810	1:20
Anti-human CD3	APC/Cy7	UCHT1	BioLegend	300426	1:20
Anti-human CD14	Pacific Blue	M5E2	BioLegend	301828	1:20
Anti-human HLA-DR	BV510	L243	BioLegend	307646	1:20

Table S2. The table lists all monoclonal antibodies and reagents used for flow cytometric staining, including fluorochrome conjugates, clone identifiers, manufacturers, catalog numbers, and staining dilutions.
